# Pseudodominant AOA2

**DOI:** 10.1186/s40673-015-0024-0

**Published:** 2015-04-30

**Authors:** Laurence Newrick, Malcolm Taylor, Marios Hadjivassiliou

**Affiliations:** Great Western Hospital, Swindon, UK; School of Cancer Sciences, University of Birmingham, Birmingham, UK; Department of Neurology, Royal Hallamshire Hospital, Sheffield, UK

**Keywords:** Cerebellar, Ataxia, Ataxia with occulomotor apraxia type 2

## Abstract

We report a mother and daughter with autosomal recessive ataxia with occulomotor apraxia in whom sequence analysis of senataxin revealed a dignosis of AOA2 (ataxia with occulomotor apraxia type 2) in both individuals. The apparent dominant inheritance pattern (pseudodominant) was the result of the unusual coincidence of both mother and daughter being compound heterozygotes for senataxin mutations. Our case exemplifies the challenges of diagnosis in hereditary ataxias, and the limitations of genetic testing guided solely by patterns of inheritance.

## Background

Ataxia with occulomotor apraxia type 2 (AOA2) is a rare autosomal recessive cerebellar ataxia caused by mutations in the senataxin gene (*SETX*) [1]. It is characterised by a progressive early-onset cerebellar ataxia, sensorimotor peripheral neuropathy and variably present occulomotor apraxia [2]. Raised alpha-feto-protein (AFP) and cerebellar atrophy are typical. The diagnosis of hereditary ataxias is often problematic [3]. Targeted genetic testing is guided by the phenotype, in which the pattern of inheritance is crucial. We report the unusual case of pseudodominance in a family with autosomal recessive AOA2.

## Case presentation

A mother (Ia) and daughter (IIa) with a presumed autosomal dominant early-onset cerebellar ataxia were reviewed in the Sheffield (UK) Ataxia Clinic. The 47 year old female IIa had a longstanding history of cerebellar ataxia spanning three decades. She had initially presented with incoordination and diplopia aged 11. Progressive deterioration in gait had resulted in the use of a wheelchair by adulthood. Age 47 she retained the ability to transiently weight bear, with a severely ataxic gait on assisted walking. On examination she had signs of cerebellar dysfunction including limb and truncal ataxia, dysarthria, intention tremor, dysdiadokinesis and dysmetric finger pointing bilaterally. Horizontal and vertical gaze-evoked nystagmus and dysmetric saccades were present, without occulomotor apraxia. Symmetrical distal weakness and wasting were marked, with generalised areflexia. Plantar responses were normal. Sensory deficits included impaired vibration and pinprick sensation in the lower limbs and left forearm. Telangiectasia was not evident. She did not suffer from frequent infections and there was no history of cancer in her or her family. MRI demonstrated cerebellar and mild supratentorial atrophy. Neurophysiology showed a length dependent axonal sensorimotor peripheral neuropathy. AFP was elevated on repeated testing ranging from 63–72 KU/l (normal range 0-15KU/L). Immunoglobulins and vitamin levels were normal. The mother of the index patient (Ia) shared a similar phenotype of progressive early-onset cerebellar ataxia (in her teens), severe length dependent axonal polyneuropathy, elevated AFP (149KU/L), and cerebellar atrophy. She presented to the respiratory physicians at the age of 65 because of respiratory difficulties. She was wheelchair bound with severe distal muscle wasting. Investigations suggested that her respiratory problems were secondary to her severe neuropathy. Although disabled for many years she was never investigated for her neurological problems prior to her respiratory problems. Mother and daughter had no contact with the biological father of IIa. There were no other siblings and thus no further family history was available.

### Genetic analysis

Both patients underwent genetic testing for Friedreich’s ataxia and common Spinocerebellar ataxias (SCA1, 2, 3, 6, 7) which were negative. In view of the unusual combination of an apparently dominantly-inherited cerebellar ataxia with a raised AFP, DNA was sent for further genetic analysis. Sequencing of the entire coding region of *SETX* identified two pathogenic mutations in patient IIa confirming the diagnosis of AOA2, including: c.5274 + 4_5274 + 7del with a predicted effect on splicing and c.6686_6687insCAAC; p.Met2229Ilefs*43 which may result in loss of expression through nonsense mediated decay or a truncated protein. c.5274 + 4 5274 + 7del was inherited from the mother (Ia) who was also a compound heterozygote with a second likely loss of function mutation (c.7121_7122delTG; p. Val2374Glyfs*20). These *SETX* mutations are not present in the LOVD UCL Neurogenetics *SETX* database, although both the c.6686_6687insCAAC and c.7121_7122delTG *SETX* mutations have been identified in previous cases of AOA2 in the UK. It is unclear whether c.6686_6687insCAAC represents a de novo mutation or was inherited paternally. Unfortunately genetic material was unavailable from the estranged father.

## Conclusions

The diagnosis of hereditary ataxias is often challenging [3]. There is substantial overlap in the phenotypes of these genetically distinct disorders with significant variability in their presentation. Given the apparent autosomal dominant pattern of inheritance the family were tested for the common autosomal dominant ataxias (SCA1, 2, 3, 6, 7). The unusual combination of an elevated AFP in an apparent ‘autosomal-dominant’ ataxia with axonal neuropathy led to the consideration of an atypically inherited AOA2. Raised AFP is not seen in other ataxias associated with severe axonal neuropathy (eg SCA4, 18, 25, AOA1 and SCAN) [4]. Furthermore, the occurrence of cerebellar atrophy and severe neuropathy in the context of normal immune function and immunoglobulins, absent telangiectasia and onset in the second decade would be more suggestive of AOA2 than Ataxia Telangiectasia. Occulomotor apraxia may be absent in approximately 50% of patients [2]. Misleading patterns of inheritance in AOA2 have been previously reported [5,6]. In this case, the false categorisation of AOA2 as an autosomal dominant ataxia was explained by the unusual coincidence of a mother (Ia) with AOA2 who was a compound heterozygote for 2 *SETX* mutations having a daughter (IIa) with AOA2 who was also a compound heterozygote for mutations in *SETX*.

The absence of paternal genetic material means we are unable to clarify the source of patient IIa’s additional SETX mutation (de novo or paternally inherited). Pseudodominance is often observed in the context of consanguinity, although no such history was elicited in this family.

The correlation between genotype and phenotype in AOA2 remains unclear. It has been proposed that mutations in the *SETX* helicase domain, particularly missense, may be associated with a mildly attenuated phenotype characterised by slower progression compared with other *SETX* sites [2]. However, the clinical phenotype of AOA2 remains relatively homogenous in most series. Interestingly, although fertility has not been directly studied, hypogondatrophic hypogonadism has been reported in 2 female patients with AOA2 in a previous cohort [2]. The present case report confirms the reproductive fitness of at least some individuals with AOA2.

Considering the continually expanding number of genetic mutations associated with hereditary ataxias, molecular genetic diagnosis is often never achieved or done so after a lengthy time period. Algorithms for genetic testing sometimes rely on panels targeted at specific patterns of inheritance, particularly for autosomal dominant families [3]. The present case highlights the limitations of such an approach, especially when applied without considering additional clinical information. Next generation sequencing (NGS) is already offering a more comprehensive and rapid molecular genetic evaluation of potentially inherited diseases. Interestingly, such ataxia panel tests include both autosomal dominant and recessive ataxias [7].

## Consent

The patients have given consent for the report to be published.

## Abbreviations

AOA1: Ataxia with occulomotor apraxia type 1
AOA2: Ataxia with occulomotor apraxia type 2
AFP: Alpha-feto-protein
DNA: Deoxyribonucleic acid
SCA: Spinocerebellar ataxia
SETX: Senataxin gene
SCAN: Spinocerebellar ataxia with axonal neuropathy
